# Genomic selection strategies to increase genetic gain in tea breeding programs

**DOI:** 10.1002/tpg2.20282

**Published:** 2022-11-09

**Authors:** Nelson Lubanga, Festo Massawe, Sean Mayes, Gregor Gorjanc, Jon Bančič

**Affiliations:** ^1^ The Roslin Institute and Royal (Dick) School of Veterinary Studies The Univ. of Edinburgh Easter Bush Campus Midlothian EH25 9RG UK; ^2^ School of Biosciences The Univ. of Nottingham Malaysia Jalan Broga Semenyih Selangor Darul Ehsan 43500 Malaysia; ^3^ School of Biosciences The Univ. of Nottingham Sutton Bonington Campus Loughborough Leicestershire LE12 5RD UK

## Abstract

Tea [*Camellia sinensis* (L.) O. Kuntze] is mainly grown in low‐ to middle‐income countries (LMIC) and is a global commodity. Breeding programs in these countries face the challenge of increasing genetic gain because the accuracy of selecting superior genotypes is low and resources are limited. Phenotypic selection (PS) is traditionally the primary method of developing improved tea varieties and can take over 16 yr. Genomic selection (GS) can be used to improve the efficiency of tea breeding by increasing selection accuracy and shortening the generation interval and breeding cycle. Our main objective was to investigate the potential of implementing GS in tea‐breeding programs to speed up genetic progress despite the low cost of PS in LMIC. We used stochastic simulations to compare three GS‐breeding programs with a Pedigree and PS program. The PS program mimicked a practical commercial tea‐breeding program over a 40‐yr breeding period. All the GS programs achieved at least 1.65 times higher genetic gains than the PS program and 1.4 times compared with Seed‐Ped program. Seed‐GSc was the most cost‐effective strategy of implementing GS in tea‐breeding programs. It introduces GS at the seedlings stage to increase selection accuracy early in the program and reduced the generation interval to 2 yr. The Seed‐Ped program outperformed PS by 1.2 times and could be implemented where it is not possible to use GS. Our results indicate that GS could be used to improve genetic gain per unit time and cost even in cost‐constrained tea‐breeding programs.

## Core Ideas

- Genomic selection strategies achieved higher genetic gains than phenotypic and pedigree‐based selection programs.
- Cost‐constrained Seedlings Genomic Selection was the most promising and cost‐effective strategy.
- The Seedlings Genomic Selection program introduced genomic selection at the seedlings stage and had 1.7 times more genetic gain than phenotypic selection.
- The pedigree program outperformed phenotypic selection and can be implemented in cases where genomic selection cannot be used.

**Abbreviations**
ACT: advanced clonal trial stage
BLUP: best linear unbiased prediction
ECT: elite clonal trial stage
ECT‐GS: elite clonal trials genomic selection breeding program
GS: genomic selection
LMIC: low‐ to middle‐income countries
PS: phenotypic selection
QTL: quantitative trait locus
Seed‐GSc: cost‐constrained seedlings genomic selection breeding program
Seed‐GSunc: cost‐unconstrained seedlings genomic selection breeding program
Seed‐Ped: seedlings pedigree breeding program

## 1 INTRODUCTION

Tea [*Camellia sinensis* (L.) O. Kuntze] is mainly grown in tropical and subtropical regions in low‐ to middle‐income countries (LMIC) (Mondal, 2014). It is an important crop for the economies of these countries as it provides a source of income for many smallholder farmers and those employed in tea‐processing companies (Wambulwa et al., 2021). Additionally, the tea‐growing areas have benefitted from improved social infrastructure such as good road networks, schools, and hospitals. All tea varieties currently grown in the world originated in China and India and were either directly or indirectly imported from these two countries to other countries (Meegahakumbura et al., 2018; Wambulwa et al., 2021). As the world population increases, it is expected that the demand for beverages will also increase (Valin et al., 2014). Conventional tea breeding is well established in the major tea‐growing countries such as China, India, and Kenya and has led to the development of many superior varieties (Chen et al., 2012). In Kenya, Tea Research Institute has in the past released high yielding and good quality varieties such as TRFK 31/8 and TRFK 303/577 and TRFK 6/8 (Kamunya et al., 2012). These varieties are widely cultivated by most smallholders and the main multinational tea companies in Kenya. To sustain long‐term tea production and the increasing demand for tea, breeders need to continuously bring new improved varieties to the market. Tea‐breeding goals vary among the major tea‐growing countries, depending on local needs. However, in the recent times, the most important tea‐breeding objectives are to develop varieties with high yield and improved quality (i.e. color, aroma, taste, and mouthfeel) (Kamunya et al., 2012; Mondal, 2014). Currently, tea productivity is seriously threatened by climate change, which is already causing yield losses and decreased quality (Gunathilaka et al., 2017). Climate change has led to extreme and unpredictable weather patterns, resulting in longer dry spells, heavy rainfall, more hail, higher temperatures, and increased attacks of pests and diseases (Marx et al., 2017). Therefore, effective tea‐breeding strategies that use genomic‐assisted breeding are needed to develop high‐yielding and high‐quality tea varieties that are also tolerant to biotic and abiotic stresses (Mondal, 2011; Muoki et al., 2020).

**FIGURE 1:**
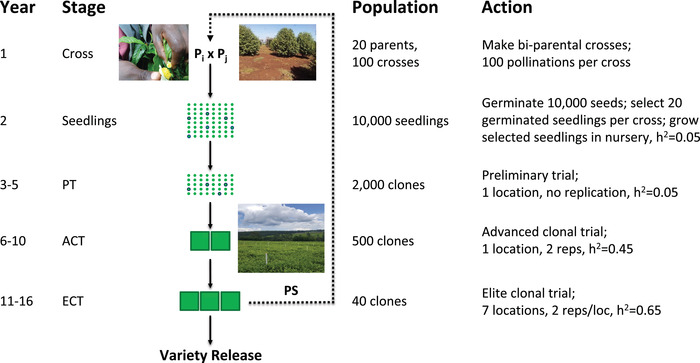
Schematic overview of the phenotypic selection breeding program (PS). This program is based on a commercial tea breeding program as described by Carr (2018). The dashed line represents the stage at which the 5 or 20 new parents are selected based on phenotypic information. PT, Preliminary Trial stage; ACT, Advanced Clonal Trial stage and ECT, Elite Clonal Trial stage

Tea‐breeding programs traditionally use recurrent phenotypic selection (PS) to identify the best individuals based on phenotypic values estimated from the per se performance of clones in evaluation trials. This involves the creation of genetic variation through crossing, followed by many years of testing to determine the genetic value of promising genotypes, leading to the identification of genotypes that will serve as new parents for crossing and for the commercial release (Kamunya et al., 2012). In the initial phase of the breeding program, new genotypes are first tested as seedlings in single bush (preliminary) trials. Then, selected seedlings are clonally propagated allowing the clones to be tested across multiple locations and years (Carr, 2018). The PS has been somewhat successful in delivering improved tea varieties over many years (Mondal, 2014). However, it is a time‐consuming process as it takes about 16 yr to develop new varieties for commercial release (Figure 1).

**Core Ideas**

- Genomic selection strategies achieved higher genetic gains than phenotypic and pedigree‐based selection programs.
- Cost‐constrained Seedlings Genomic Selection was the most promising and cost‐effective strategy.
- The Seedlings Genomic Selection program introduced genomic selection at the seedlings stage and had 1.7 times more genetic gain than phenotypic selection.
- The pedigree program outperformed phenotypic selection and can be implemented in cases where genomic selection cannot be used.

Pedigree best linear unbiased prediction (BLUP) method predicts estimated breeding values (EBVs) using information from known genetic relationships among the parents and available phenotypic data on related crosses (Bernardo, 1996; Piepho et al., 2008). The BLUPs are obtained by defining the genotypes as random effects in a mixed linear model (Henderson, 1975). To our knowledge, most tea‐breeding programs traditionally do not use pedigree‐based selection although inclusion of genetic relationships can improve selection accuracy. In soybean [*Glycine max* (L.) Merr.], the application of pedigree BLUP resulted in more superior crosses that were selected compared with the traditional mid‐parent value (Panter & Allen, 1995). Similarly, Bernardo (1996) used Pedigree BLUP to predict performance of untested single crosses of maize (*Zea mays* L.) using relationship data from relatives. In forest breeding, pedigree BLUP based on kinship relationships between individuals is commonly used to identify superior trees with traits of interest (Alves et al., 2018). However, pedigree‐based relationships do not account for random Mendelian sampling and therefore within‐family selection is not possible (Hill & Weir, 2011).

In modern times, plant breeding has started to move from complete reliance on PS to genomic‐assisted selection due to improved molecular biology and high‐throughput genotyping technologies (Leng et al., 2017). Genomic selection (GS) offers a great potential for more accurately identifying good parents and superior clones for variety development and commercial release, and thus increasing the genetic gain of tea‐breeding programs (Meuwissen et al., 2001). In GS, molecular markers are used to estimate relatedness between individuals and represent realized genomic relationships rather than the expected relationships. This makes it possible to capture distant relationships and variation in sibling relationships due to Mendelian sampling, leading to more accurate estimates of additive genetic variance and breeding values (Legarra et al., 2009). In tree breeding, GS has been reported to have a substantial impact on the rate of genetic gains due to the increased accuracy in estimating the additive genetic variance and the breeding values (Grattapaglia et al., 2018). In some animal‐breeding programs, the introduction of GS has resulted in almost doubled genetic progress compared with pedigree‐based selection (García‐Ruiz et al., 2016).

Genomic selection involves estimation of genomic estimated breeding values (GEBVs) by summing marker effects that are in linkage disequilibrium with one or more quantitative trait loci (QTLs) across the entire genome (Bernardo & Yu, 2007). Genomic selection uses a prediction model that is first trained using a population of genotyped and phenotyped individuals and is then used to predict GEBVs of selection candidates with genotyping information but no phenotypes. This is especially useful for key tea traits such as tea yield and quality, which are controlled by many genes with small effects and the use of traditional marker‐assisted selection is limited (Bernardo & Yu, 2007). By then correlating GEBVs with the actual phenotypic data, it is possible to assess the accuracy of the GS model (Heffner et al., 2009; Meuwissen et al., 2001). For instance, Lubanga et al. (2021) investigated the potential use of GS to improve tea quality and reported higher prediction accuracies for all genomic prediction models compared with the Pedigree BLUP model. Similar findings were also reported by Yamashita et al. (2020), who also investigated the potential of using GS to improve tea quality and they found moderate prediction accuracies for the six tested genomic prediction models.

In practice, GS can be used to improve the rate of genetic gain and accelerate the release of new improved varieties in a tea breeding programs by:

1. Reducing the generation interval since new parents can be selected at the earliest Seedlings stage rather than in late testing stages.
2. Increasing the accuracy of selecting superior tea genotypes at the Seedlings stage where selection accuracy is generally very low.
3. Increasing the selection intensity at the nursery stage by testing more genotyped seedlings compared with PS.
4. Shortening the entire breeding cycle time by reducing or eliminating lengthy field progeny testing.

The implementation of GS in LMIC faces limitations, however. In most of these countries, the cost of phenotypic selection is much lower compared with Europe and North America because the local population provides labor at a lower cost. In addition, most breeding programs in LMIC have limited investment budgets for conducting research and there are few qualified personnel who understand GS and its practical implementation in breeding programs. Therefore, the possibility of successful implementation of GS in tea breeding should be empirically tested.

Plant breeders have traditionally relied on lengthy and costly field trial experiments to inform their decisions (Ahmar et al., 2021). Stochastic simulations are useful in identifying promising breeding strategies through studying the genetic gain, predictive accuracy, and cost‐effectiveness of GS under different scenarios (Gaynor et al., 2021). Simulations have been conducted for many crops, including wheat (*Triticum aestivum* L.) (Gaynor et al., 2017), maize (Powell et al., 2020), sorghum [*Sorghum bicolor* (L.) Moench] (Muleta et al., 2019), clonally propagated crops (Werner et al., 2020), and trees (Iwata et al., 2011). However, to our knowledge, no simulation study has been published integrating GS into a tea‐breeding program to investigate the feasibility and long‐term outcomes.

Our study aims to test the feasibility of implementing GS in a tea‐breeding program using stochastic simulations. A phenotypic selection breeding program was used as a baseline in which the number of crosses, seedlings, replicates, and locations mimicked a practical commercial tea‐breeding program. Using AlphaSimR R software, we developed three new breeding programs that integrated GS and a pedigree selection program and compared them to the PS‐breeding program. Our objectives were to (a) investigate the potential of implementing GS in tea‐breeding programs despite the limited resources and low cost of PS in LMIC, (b) compare different strategies of implementing GS in tea‐breeding programs at the same cost as PS, and (c) quantify how shortening the tea‐breeding generation interval and the entire breeding cycle length using GS leads to higher genetic gains.

## 2 MATERIALS AND METHODS

We used stochastic simulations to evaluate the possibility of implementing GS in tea‐breeding programs. We compared three breeding strategies incorporating GS to a pedigree‐based and a commercial PS‐breeding program. We subdivided the Materials and Methods section into simulation of the founder genotype population and simulation of the breeding programs.

We simulated the founder genotype population as follows:

1. Genome simulation: A genome sequence was simulated for a hypothetical diploid tea species.
2. Simulation of founder genotypes: The simulated genome sequences were used to generate a base population of 20 diploid founder genotypes.
3. Simulation of genetic values: A single trait representing yield was simulated for all founder genotypes by summing the additive effects at 2,400 quantitative trait nucleotides (QTN).
4. Simulation of phenotypes: The phenotypes of all founder genotypes were simulated by adding random error to the total genetic value of the tea genotypes.

We simulated the breeding programs in two phases as follows:

1. Recent (burn‐in) breeding phase: A PS‐breeding program was simulated for a period of 40 yr (burn‐in) to provide a common starting point for all breeding programs in the future breeding phase.
2. Future breeding phase: A pedigree and three different GS‐breeding programs were simulated and compared with the PS‐breeding program for a period of 40 yr.

### 2.1 Simulation of the founder genotype population

#### 2.1.1 Genome simulation

We simulated a genome sequence with 15 pairs of chromosomes to reflect a hypothetical diploid tea species [*Camellia sinensis* (L.) O. Kuntze]. Each chromosome was assigned a physical length of 10^8^ base pairs and a genetic length of 1 Morgans. The chromosome sequences were generated using the Markovian coalescent simulator (MaCS) (Chen et al., 2009), implemented in AlphaSimR (Gaynor et al., 2021). The recombination rate was set to 10^−8^, mutation rate to 2.5 × 10^−8^ and effective population size (Ne) to 100 in the base population as in Werner et al. (2020).

#### 2.1.2 Simulation of founder genotypes

We used the simulated genome sequences to generate a base population of 20 diploid founder genotypes by randomly sampling 15 chromosome pairs per genotype. A set of 160 biallelic quantitative trait nucleotides (QTNs) and 600 single nucleotide polymorphisms (SNPs) were randomly selected along each chromosome to simulate a quantitative trait controlled by 2,400 QTN and a SNP marker array with 9,000 genome‐wide SNP markers.

#### 2.1.3 Simulation of genetic values

We simulated genetic values for a single trait by summing the additive genetic effects of 2,400 sampled QTNs. Additive genetic effects (*a*) were sampled from the standard normal distribution and scaled them to obtain an additive genetic variance of $\sigma_{a}^{2}=150,000$in the founder population. Each year, additive genetic effects were also scaled by a single environmental covariate value to simulate the genotype × year (G×Y) effect (Gaynor et al., 2021). The environmental covariates were sampled from the normal distribution with mean 0 and variance was a square root of$\sigma_{G\times Y}^{2}$, which was assumed to be equal to $\sigma_{a}^{2}$.

#### 2.1.4 Simulation of phenotypes

We simulated a single trait representing tea yield (measured in kilogram made tea per hectare; kg MT ha^–1^) by adding random error to additive genetic value. The random error was sampled from the standard normal distribution with mean zero and variance $\sigma_{e}^{2}$. The $\sigma_{e}^{2}$ was defined by the target level of narrow‐sense heritability (ℎ^2^) at each testing stage of the tea‐breeding program. In the founder population, we calculated the entry‐mean values obtained from previous studies by Lubanga et al. (2021) to determine narrow‐sense heritability (ℎ^2^) at each breeding stage. The ℎ^2^ at the Seedling and PT stages was .05, .45 in the advanced clonal testing stage (ACT) and .65 in the elite clonal testing (ECT) stage. The heritabilities in later testing stages were higher because of the increased number of replicates per genotype. Narrow‐sense heritability was calculated as

$$\frac{\sigma_{a}^{2}}{\left(\sigma_{a}^{2}+\sigma_{G\times Y}^{2}/e+\sigma_{e}^{2}/er\right)},$$

where *e* and *r* are the number of environments and replicates within each environment, respectively.

### 2.2 Recent (burn‐in) breeding phase

We simulated the burn‐in phase over a 40‐yr period using a PS program to establish a common baseline for the future breeding phase. The structure of the PS program was based on a practical commercial tea‐breeding program (Figure 1), and a more detailed description can be found in Carr (2018). Prior to the start of the burn‐in phase, 16 crossings and selection cycles were conducted by crossing the same 20 founder genotypes to fill the breeding pipeline with unique genotypes at each stage. After the initial filling stage, the burn‐in phase continued for 40 yr. Twenty parental genotypes were crossed each year to make 100 bi‐parental crosses with 100 pollinations per cross to achieve a total of 10,000 crosses. Based on our experience, we assumed that about 20% (i.e., 2,000) seedlings germinated and were grown in the nursery for 1 yr. The following year, seedlings were planted in the field as preliminary trials (PT), followed by a 3‐yr evaluation period. Five hundred superior clones were selected and planted in ACT, and yield data were recorded for 5 yr. Forty clones with the highest yields were then advanced to the ECT, and yield data were recorded for 6 yr until a variety was selected for release. Each year, new parents were selected at the ECT stage in Year 16, and the five oldest parents in the crossing block were replaced with the new high‐yielding genotypes from the ECT stage. The total breeding cycle duration of the PS breeding program was 16 yr (Figure 1).

### 2.3 Future breeding phase

In the future breeding phase, a pedigree‐based and three GS programs were compared with the PS program. Each breeding program used the burn‐in phase as a starting point and was tested for 40 yr. The operating costs of the three GS strategies are presented in detail in Table 1 and were equated to the estimated cost of the PS program (US$71,880). The pedigree‐based program was assumed to have the same costs as the PS program. The equalization of costs in the GS programs was based on the estimated costs of genotyping and reducing program sizes at different breeding stages. We assumed that the cost of genotyping per individual was $15 (e.g. https://excellenceinbreeding.org/toolbox/services/mid-density-genotyping-service), whereas phenotyping costs were assumed to be for pollination; maintaining seedlings in the nursery; and recording data at the PT, ACT, and ECT stages in plots. The sizes and costs of the five breeding programs are shown in Table 1, and a summary of the key differences among the breeding programs is provided in Table 2.

**TABLE 1:** Summary of sizes and annual operating costs of the five simulated tea‐breeding programs

Breeding program	No. of parents	Seedlings	PT	ACT	ECT	Cost
						US$
PS	20	2,000	2,000	500	40	71,880
Seed‐Ped	20	2,000	0	500	40	71,880
Seed‐Gsc	20	800	0	300	40	69,980
Seed‐GSunc	20	2,000	0	500	40	100,980
ECT‐GS	20	800	0	0	90	72,970

*Note*. ACT, Advanced Clonal Trial stage; ECT, Elite Clonal Trial stage; ECT‐GS, Elite Clonal Trial Genomic Selection Breeding Program; GS, genomic selection; PS, phenotypic selection breeding program; PT, Preliminary Trial stage; Seed‐GSc, Cost‐constrained Seedlings Genomic Selection Breeding Program; Seed‐GSunc, Cost‐unconstrained Seedlings Genomic Selection Breeding Program; Seed‐Ped, Seedlings Pedigree Breeding Program.

**TABLE 2:** Summary of the key differences between the five simulated tea‐breeding programs

Program	Parent selection stage	Parent selection	Generation interval/breeding cycle duration	Key features
			yr	
PS	ECT	Phenotype	16/16	Conventional breeding
Seed‐Ped	Seedlings	Pedigree	2/13	PT stage removed, selection from 2,000 seedlings
Seed‐GSc	Seedlings	GS	2/13	PT stage removed; 800 seedlings genotyped
Seed‐GSunc	Seedlings	GS	2/13	PT stage removed; 2,000 seedlings genotyped
ECT‐GS	Seedlings	GS	2/8	PT and ACT stages removed; increased number of clones tested in ECT stage

*Note*. ACT, Advanced Clonal Trial stage; ECT, Elite Clonal Trial stage; ECT‐GS, Elite Clonal Trial Genomic Selection Breeding Program; GS, genomic selection; PS, phenotypic selection breeding program; PT, Preliminary Trial stage; Seed‐GSc, Cost‐constrained Seedlings Genomic Selection Breeding Program; Seed‐GSunc, Cost‐unconstrained Seedlings Genomic Selection Breeding Program; Seed‐Ped, Seedlings Pedigree Breeding Program.

Additionally, two parent replacement methods were tested for each of the five breeding programs by:

1. Replacing 25% (5) parents after each breeding cycle.
2. Replacing 100% (20) parents after each breeding cycle.

A common burn‐in was used for both methods to ensure that the results were directly comparable.

#### 2.3.1 Seedlings Pedigree Breeding Program

**FIGURE 2:**
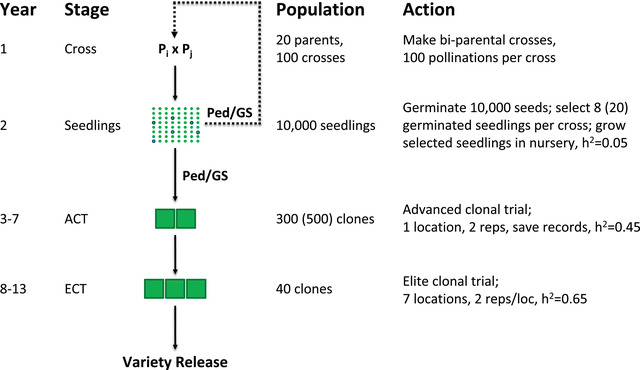
Schematic overview of the Pedigree Breeding Program (Seed‐Ped) and Seedlings Genomic Selection Breeding Program with constrained costs (Seed‐GSc) and unconstrained costs (Seed‐GSunc). The dashed line represents the stage at which the 5 or 20 new parents are selected based on genomic prediction or pedigree best linear unbiased prediction (BLUP). The values outside the brackets refer to Seed‐GSc and the values inside the brackets refer to Seed‐GSunc. Seed‐Ped, Seedlings pedigree selection; GS, genomic selection; ACT, Advanced Clonal Trial stage; and ECT, Elite Clonal Trial stage

The Seedlings Pedigree Breeding Program (Seed‐Ped program) incorporated known genetic relationships among the parents and the available phenotypic data of the crosses to predict EBVs for tea yield. These were obtained from the pedigree BLUP model to advance superior genotypes to the next stage and select the best 5 or 20 genotypes to replace the oldest parents in the crossing block. The PT stage was eliminated to avoid 3 yr of field trial testing. The EBVs were used to advance the best 300 genotypes from the Seedlings stage to the ACT stage. Yield trials were recorded at the ACT stage for 5 yr. Forty promising clones with the highest EBVs were advanced to the ECT stage and yield trials were recorded for 6 yr until variety release. This program had a 2‐yr generation interval and lasted a total of 13 yr, which is 3 yr shorter than the PS breeding program (Figure 2).

#### 2.3.2 Cost‐constrained Seedlings Genomic Selection Breeding Program

The Seedlings Genomic Selection Breeding Program (Seed‐GSc program) has a similar strategy to the Seed‐Ped program, except that genotyping and GS were introduced at the Seedlings stage. The preliminary trial (PT) stage was also eliminated (Figure 2). Eight hundred (eight per family) seedlings were randomly selected for genotyping from the 2,000 germinated seeds in the nursery due to budget constraints. Genomic selection was used to advance the genotypes to the next stage and select the best 5 or 20 genotypes to replace the oldest parents in the crossing block. The PT stage was eliminated to avoid 3 yr of field trial testing, and therefore GS was used to advance the best 300 genotypes from the Seedlings stage to the ACT stage. Yield trials were recorded at the ACT stage over 5 yr. Forty promising clones with the highest GEBVs were advanced to the ECT stage and yield trials were recorded for 6 yr until variety release. The Seed‐GSc program had a 2‐yr generation interval and lasted a total of 13 yr, which is 3 yr shorter than the PS‐breeding program.

#### 2.3.3 Cost‐unconstrained Seedlings Genomic Selection Breeding Program

The Cost‐unconstrained Seedlings Genomic Selection Breeding Program (Seed‐GSunc program) followed the same strategy as the Seed‐GSc program, except that it used a higher operating budget (Figure 2). There are two main differences between the two programs: (a) all 2,000 seedlings (instead of 800) at the Seedlings stage were genotyped and predicted using GS, and (b) GS was used to advance a total of 500 genotypes (instead of 300) to the ACT stage. The Seed‐GSunc program also had a 2‐yr generation interval and lasted a total of 13 yr, which is 3 yr shorter than the PS‐breeding program.

#### 2.3.4 Elite Clonal Trial Genomic Selection Breeding Program

**FIGURE 3:**
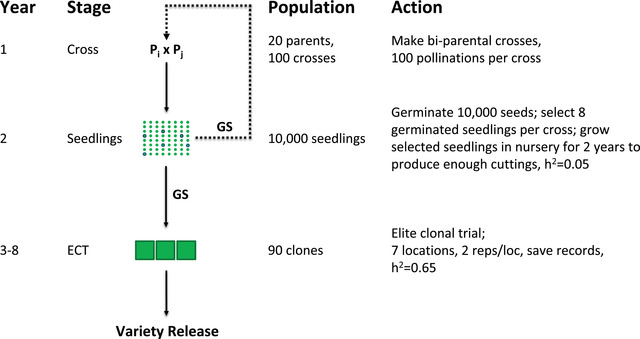
Schematic overview of the Elite Clonal Trial Genomic Selection Breeding Program (ECT‐GS). The dashed line represents the stage at which the 5 or 20 new parents are selected based on genomic prediction. GS, genomic selection; ACT, Advanced Clonal Trial stage; and ECT, Elite Clonal Trial stage

The Elite Clonal Trial Genomic Selection Breeding Program (ECT‐GS program) introduced genotyping and GS at the earliest Seedlings stage and eliminated the PT and ACT stages (Figure 3). The two stages were eliminated to avoid 8 yr of testing and to reallocate the resources for genotyping all 2,000 seedlings at the Seedlings stage as in the PS program. Compared with the previous two GS programs, seedlings were planted in the nursery for an additional year to produce enough cuttings for direct planting at the ECT stage. The GS was then used to advance genotypes to the next stage and select the best 5 or 20 genotypes to replace the oldest parents in the crossing block. Ninety promising clones were advanced from the Seedlings stage to the ECT stage, where they were evaluated for 6 yr until variety release. The ECT‐GS breeding program has a 2‐yr generation interval and lasts a total of 8 yr, which is 8 yr shorter than the PS‐breeding program.

### 2.4 Training population and models

Pedigree and phenotypic data were collected from the ACT stage during the last 6 yr of the burn‐in phase and new data were added to the training population as new yield trials were recorded in subsequent years. The initial training population consisted of 3,000 phenotypic records from the ACT genotypes. After 40 yr of future breeding, the training population increased to 23,000 records. The construction of the pedigree relationship matrix and fitting of the pedigree BLUP model was performed using the ASReml‐R v4.1 R package (Butler et al., 2017). Year was fitted as a fixed effect to account for G×Y effect and heterogeneous error variance was allowed for each year.

To initialize the GS training population, phenotypic data were collected from the ACT stage in the last 6 yr of the burn‐in phase. The initial training population consisted of 3,000 phenotypic records from the ACT genotypes and new data were added to the training population as new yield trials were recorded in subsequent years. For the Seed‐GSunc and Seed‐GSc programs, ACT data were used to update the training population, whereas only ECT data were used to update the ECT‐GS program. After 40 yr of future breeding, the training population increased to 23,000 (Seed‐GSunc, Seed‐GSc), and 6,600 (ECT‐GS) records. A ridge regression best linear unbiased prediction model (RR‐BLUP) was used for genomic predictions (Meuwissen et al., 2001). Year was fitted as a fixed effect to account for G×Y effect and heterogeneous error variance was allowed for each year. The predicted additive SNP effects at each marker locus were then multiplied with genotypes and summed to obtain GEBVs.

### 2.5 Evaluation and comparison of the tea‐breeding programs

The efficacy of the Seed‐Ped and the three GS‐breeding programs was compared with the PS program by tracking the mean genetic values of the newly developed genotypes at the Seedlings stage over a 40‐yr period in the future phase. Genetic variance, selection accuracy, and the efficiency of converting genetic diversity into genetic gain were also tracked for all breeding programs at the Seedlings stage. All genetic parameters were tracked at the Seedlings stage, as this is the earliest stage at which all programs evaluated new crosses (F1 seedlings). Genetic gain and variance were assessed by plotting the mean and variance of the Seedlings population genetic values against time. We calculated the prediction accuracy for the GS‐breeding programs as the correlation between the true genetic values and their GEBVs at the Seedlings stage. The efficiency of converting genetic diversity into genetic gain was measured by regressing achieved genetic gain on lost genetic diversity, as described by Gorjanc et al. (2018). All simulations for each strategy were repeated 30 times.

Comparison between breeding programs was expressed as ratios. These were calculated by applying the paired Welch test to the log transformed values of the 30 simulation replicates. The log‐transformed differences from *t* tests were then back‐transformed to obtain the ratios (Ramsey & Schafer, 1997). Significant differences between mean genetic values from the final year between all pairwise combinations of the five breeding programs were determined using Tukey's HSD test (*p* < .05).

## 3 RESULTS

Our simulations showed that the Seed‐Ped and all the GS‐breeding programs outperformed PS program. The Seed‐GSunc program had the highest overall genetic gain. All GS‐breeding programs had higher selection accuracies compared with the Seed‐Ped and PS‐breeding programs. Genetic variance decreased rapidly over time for the GS programs. Replacing all parents resulted in a slightly higher genetic gain, with a slight decrease in genetic variance compared with replacing 25% of parents. The PS‐breeding program had the highest conversion efficiency, but the lowest genetic gain compared with the Seed‐Ped and GS programs.

### 3.1 Genetic gain

**FIGURE 4:**
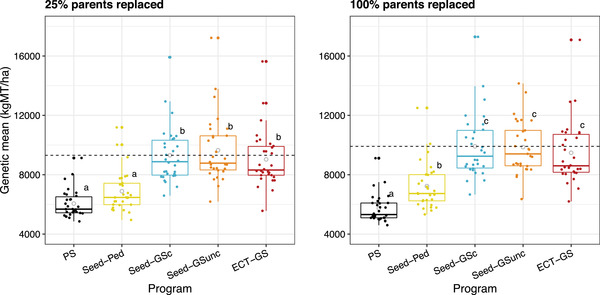
Genetic gain in the final year of the five breeding programs when 25% (left) or 100% (right) of parents were replaced. Within each boxplot, the dots represent the outcome of each simulation replicate, and the line and gray circle represent the median and mean of the 30 replicates. The dotted line represents the mean of the Seedlings Genomic Selection Breeding Program with constrained costs (Seed‐GSc) for reference

Our results showed that all the genomic selection strategies performed significantly better than Seed‐Ped and PS programs (*p* < .05) in Year 40 (Figure 4). When 25% of the parents were replaced the Seed‐Ped and PS programs were not significantly different from each other (*p* < .05). However, when all the parents were replaced, the Seed‐Ped program performed significantly better than the PS program (*p* < .05). All the GS programs were not significantly different from each other when all parents were replaced and also when 25% of the parents were replaced (*p* < .05) (Figure 4).

**FIGURE 5:**
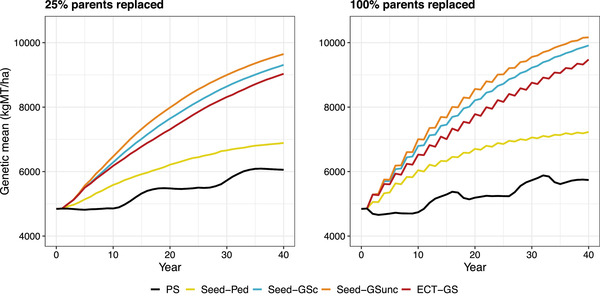
Genetic gain over time for the five breeding programs when 25% (left) or 100% (right) of parents were replaced. The lines in each plot represent the five programs, with each line representing the mean genetic values over 40 yr. These values are based on 30 simulation replicates and were measured at the Seedlings stage. Black line represents the Phenotypic Breeding Program (PS), yellow line the Seedlings Pedigree Breeding Program (Seed‐Ped), blue line the Cost‐constrained Seedlings Genomic Selection Breeding Program (Seed‐GSc), orange line the Cost‐unconstrained Seedlings Genomic Selection Breeding Program (Seed‐GSunc) and red line the Elite Clonal Trial Genomic Selection Breeding Program (ECT‐GS)

The Seed‐Ped and three GS‐breeding programs (Seed‐GSunc, Seed‐GSc, and ECT‐GS) achieved greater genetic gain compared with the PS‐breeding program over time. This is shown in Figure 5, where the mean genetic values are plotted against the number of years of breeding at the Seedlings stage. The plot shows the trends for the mean genetic values of 30 replicates for each of the tea‐breeding programs evaluated in the future phase when 25 or 100% of the parents were replaced. Seed‐GSunc showed the greatest genetic gain compared with all other programs. Both plots show that the overall ranking of the breeding programs in terms of total genetic gain was consistent across the two proportions of parents replaced. The ranking of the breeding programs from highest to lowest mean genetic gain was: Seed‐GSunc, Seed‐GSc, ECT‐GS, Seed‐Ped, and PS.

The breeding programs in which all parents were replaced showed slightly higher genetic gain than the programs in which 25% of the parents were replaced. When all parents were replaced, the best program, Seed‐GSunc, generated 1.72 times genetic gain of the PS‐breeding program. Seed‐GSc and ECT‐GS generated 1.70 and 1.65 times the genetic gain of the PS‐breeding program, respectively. The Seed‐Ped program generated 1.20 times more genetic gain compared with the PS program. When 25% of the parents were replaced, Seed‐GSunc generated 1.60 times the genetic gain of the PS‐breeding program. On the other hand, Seed‐GSc and ECT‐GS generated 1.54 and 1.50 times the genetic gain of the PS‐breeding program, respectively. The Seed‐Ped program generated 1.16 times more genetic gain compared with the PS program (Figure 5).

### 3.2 Selection accuracy

**FIGURE 6:**
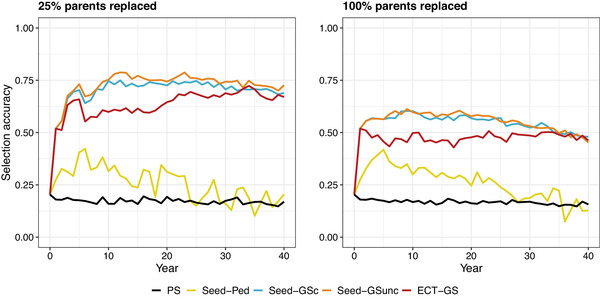
Accuracy of selection over time for the five simulated breeding programs when 25% (left) or 100% (right) of parents were replaced. The lines in each plot represent the five programs, with each line representing the mean accuracy of selection over 40 yr. These values are based on 30 simulation replicates and were measured at the Seedlings stage. Black line represents the Phenotypic Breeding Program (PS), yellow line the Seedlings Pedigree Breeding Program (Seed‐Ped), blue line the Cost‐constrained Seedlings Genomic Selection Breeding Program (Seed‐GSc), orange line the Cost‐unconstrained Seedlings Genomic Selection Breeding Program (Seed‐GSunc) and red line the Elite Clonal Trial Genomic Selection Breeding Program (ECT‐GS)

Genomic selection programs had increased selection accuracy compared with Seed‐Ped and PS programs. This is shown in Figure 6, which plots the selection accuracy as the correlation between true and estimated genetic values in the Seedlings stage across time. The plot shows the selection accuracy for all breeding programs when 25 or 100% of the parents were replaced. All GS‐breeding programs had higher selection accuracy (mean ∼.70) compared with the Seed‐Ped (mean ∼.25) and PS (mean ∼.18) ‐breeding programs. Generally, a higher selection accuracy was observed when 25% of the parents were replaced (mean ∼.50) than when all parents were replaced (mean ∼.41). Selection accuracy was stable across years for SeedGSc and SeedGSunc. In the early years of the future breeding phase, selection accuracy was lower for the ECT‐GS program compared with the other two GS‐breeding programs but then gradually increased until it reached a plateau in Year 20.

### 3.3 Genetic variance

**FIGURE 7:**
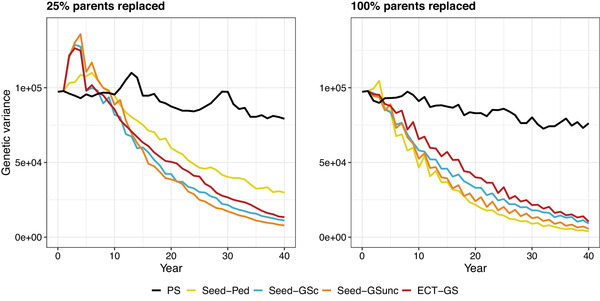
Genetic variance over time for the five simulated breeding programs when 25% (left) or 100% (right) of parents were replaced. The lines in each plot represent the five programs, with each line representing the mean genetic variance across 40 yr. These values are based on 30 simulation replicates and were measured at the Seedlings stage. Black line represents the Phenotypic Breeding Program (PS), yellow line Seedlings Pedigree Breeding Program (Seed‐Ped), blue line the Cost‐constrained Seedlings Genomic Selection Breeding Program (Seed‐GSc), orange line the Cost‐unconstrained Seedlings Genomic Selection Breeding Program (Seed‐GSunc) and red line the Elite Clonal Trial Genomic Selection Breeding Program (ECT‐GS)

Genomic selection increased the rate of loss of genetic variance compared with PS and Seed‐Ped programs. This is shown in Figure 7, which plots the genetic variance in the Seedlings stage across time. The plot shows the genetic variance for all breeding programs when 25 or 100% of the parents were replaced. All breeding programs showed a decrease in genetic variance over time. However, the rate of loss of genetic variance varied among the breeding programs. All GS‐breeding programs showed a large decrease in genetic variance (up to 99% over a 40‐yr period), whereas the PS program showed a slower decrease in genetic variance over time. Note also that the Seed‐Ped program showed a substantial loss of genetic variance, especially when all parents were replaced. The difference in genetic variance when 25 and 100% of the parents were replaced was negligible for GS programs, except during the transition period when GS was introduced and 25% of parents were replaced.

### 3.4 Conversion efficiency

**FIGURE 8:**
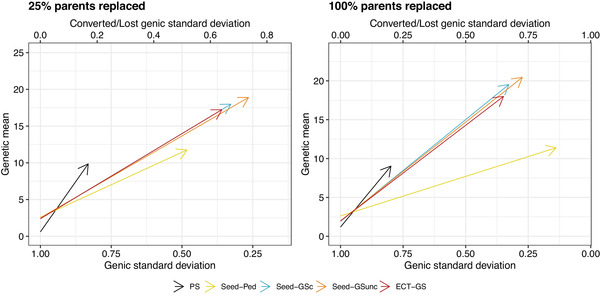
Conversion efficiency as the change in genetic mean and genic standard deviation over time for the five simulated breeding programs when 25% (left) or 100% (right) of parents were replaced. The lines in each plot represent the five programs, with the slope representing the conversion efficiency based on values from 30 simulation replicates measured at the Seedlings stage. Black line represents the Phenotypic Breeding Program (PS), yellow line the Seedlings Pedigree Breeding Program (Seed‐Ped), blue line the Cost‐constrained Seedlings Genomic Selection Breeding Program (Seed‐GSc), orange line the Cost‐unconstrained Seedlings Genomic Selection Breeding Program (Seed‐GSunc) and red line the Elite Clonal Trial Genomic Selection Breeding Program (ECT‐GS)

Breeding programs in which all parents were replaced had higher conversion efficiencies than those in which 25% of parents were replaced. This is shown in Figure 8, which plots the long‐term genetic gain in standard deviation units over long‐term change in the conversion efficiency, calculated by the slope of realized genetic gain on lost genic variance across time. In other words, the slope of the change in genetic mean over the change in the genic standard deviation quantifies the efficiency of converting genetic diversity into genetic gain. The plots show the conversion efficiency for all breeding programs when 25 or 100% of the parents were replaced.

The Seed‐GSc program had the best balance between genetic gain achieved and genetic diversity lost. When 25% of the parents were replaced, the PS‐breeding program achieved a genetic gain of 10 for a loss of .2 units of genic standard deviation (an efficiency factor of 50). The Seed‐Ped program and all GS programs had a nearly similar efficiency factor. The Seed‐GSunc program had a genetic gain of 18.50 for a loss of .75 units of genic standard deviation (an efficiency factor of 24.66). The Seed‐GSc program had a genetic gain of 18 for a loss of .70 units of genic standard deviation (an efficiency factor of 25.71). The ECT‐GS program had a genetic gain of 17.50 for a loss of .70 units of genic standard deviation (an efficiency factor of 25). The Seed‐Ped program had a genetic gain of 12 for a loss of .50 units of genic standard deviation (an efficiency factor of 24). A similar trend was observed when all parents were replaced for the PS and GS programs. The Seed‐Ped had the lowest efficiency factor, whereas the GS programs had almost the same conversion efficiency factor. The ranking of breeding programs from the highest to the lowest conversion efficiency factor was: PS (47), Seed‐GSc (30), Seed‐GSunc (28), ECT‐GS (27), and Seed‐Ped (14).

## 4 DISCUSSION

Tea‐breeding programs require the integration of genomic‐assisted breeding approaches to increase the future rate of genetic progress. However, it is currently unclear how these can be integrated into existing programs and whether the additional costs these approaches impose are feasible. In this study, we used stochastic simulations to demonstrate that the tea‐breeding programs in LMIC can benefit from GS. We developed a pedigree‐based (Seed‐Ped) and three GS‐breeding programs (Seed‐GSc, Seed‐GSunc, and ECT‐GS) and compared them to a PS‐breeding program that mimicked a practical commercial tea‐breeding program. To discuss the results, the breeding strategies are examined in terms of genetic gain, selection accuracy, genetic variance, and conversion efficiency.

### 4.1 Genetic gain

Phenotypic selection in tea breeding is an extremely slow process that requires 16 yr to develop a new tea variety. Tea is a perennial crop with a long generation interval – it takes between 3 and 6 yr for a seedling to grow into a mature bush (Mondal, 2014). To obtain reliable phenotypic information on each bush, multi‐year testing at many locations is typically required which is time‐consuming. This has resulted in slow genetic progress and release of improved varieties in tea‐breeding programs, so GS needs to be considered.

Breeding programs that used either pedigree‐based selection or GS achieved greater genetic gain compared with the PS‐breeding program. These results demonstrate the potential to improve the rate of genetic progress even in cost‐constrained tea‐breeding programs. Genomic selection was used to shorten the generation interval, increase selection accuracy, and increase selection intensity in the case of the Seed‐GSunc program. The Seed‐Ped program did not outperform any of the GS programs, but it consistently outperformed the PS program due to the shorter generation interval and improved selection accuracy as a result of the use of known genetic relationships. Shortening of the generation interval results in increased frequency with which haplotypes are recombined and exposed for selection, which increases the likelihood that superior allele combinations will emerge in newly produced progenies (Atlin et al., 2017). Similar results were previously shown by Gaynor et al. (2017) and Bančič et al. (2021) who used the advantages of GS to increase grain yield in wheat and intercrop breeding programs.

Pedigree‐based selection and GS can be used to shorten the overall length of a breeding cycle. The Seed‐Ped and GS programs therefore had between 3 and 8 yr shorter breeding cycle time compared with the PS program. Shortening may be useful for releasing superior varieties faster but can also result in lower genetic progress if done too aggressively. For example, the ECT‐GS program at Year 40 achieved a slightly lower genetic gain compared with Seed‐GSc and Seed‐GSunc likely because of the fewer number of reliable training records that could be collected in the ECT stage.

A $30,000 increase in annual operating costs was used to increase selection intensity and resulted in only slightly higher genetic gain. In the case of the Seed‐GSunc, the increased budget was used to genotype 1,200 more seedlings at the Seedlings stage as well as test more genotypes at the ACT stage compared with the Seed‐GSc program. For comparison, in the Seed‐GSc program only a subset of seedlings was selected by random sampling while still ensuring that each family contributed equally which resulted in almost similar genetic gain compared with the Seed‐GSunc. A previous study, however, showed that generating and testing more selection candidates while holding the number of selected candidates constant led to a higher selection intensity and increased the rate of genetic gain (Bernardo & Yu, 2007). Therefore, this research requires further exploration to find the most optimal point between budget increase and the change in the rate of genetic progress.

Replacement of all parents in the crossing block at the start of every breeding cycle can contribute to increased rate of genetic gain. Rapid replacement of parents increased the frequency at which haplotypes were recombined as well as increased the rate of change of frequencies for favorable alleles. The cyclic pattern observed in Figure 5 reflects this. In the PS program, parents were replaced every 16 yr, as evidenced by the two striking increases in genetic gain in both parent replacement scenarios. In the other programs, this pattern was only observed when 100% of parents were replaced in every breeding cycle. A shorter, 2‐yr generation interval in these programs results in a more frequent cyclic pattern. This was less evident when parents were replaced gradually and so changes in genetic gain were less erratic due to slower change of favorable allele frequencies.

### 4.2 Selection accuracy

Selection of promising genotypes at the Seedlings stage in tea‐breeding programs is a major limitation because selection intensity is high and selection accuracy is extremely low. In this study, broad‐sense heritabilities for the PT, ACT, and ECT stages were estimated from real data used in our previous studies (Lubanga et al., 2021). Heritability for the PT stage was estimated to be .05, for the ACT stage was .45, and for the ECT stage was .65. The low heritability at the PT stage reflects the fact that many seedlings are tested in a single unreplicated trial and selected on that basis. As a result, many promising clones are lost at this stage due to poor selection accuracy. In contrast, higher heritabilities are observed in the later stages because multi‐year and multi‐locational clonal trials with larger plots consisting of more replicates of each clone are conducted, and thus selection is more accurate. Therefore, GS was used to improve the accuracy of selection at the Seedlings stage.

Both pedigree‐based selection and GS had higher selection accuracy compared with the PS program. Pedigree‐based and genomic prediction were used at the Seedlings stage to predict the yield performance of seedlings as mature bushes more accurately to select new parents as well as advance them to the ACT stage. The higher selection accuracy was achieved by fitting the model using pedigree or genomic information on relationships and more reliable phenotypic records from the later breeding stages. The use of the late‐stage records is advantageous because genotype clones are tested repeatedly over years and across different locations compared with PS which is based on individual performance. To maintain selection accuracy in the GS programs, the training population was updated with new records each year in order to maintain a close relationship between the genotypes in the training population and selection candidates (Neyhart et al., 2017). The linkage disequilibrium (LD) between QTLs and markers is expected to change over time because of recombination, selection, and drift, which results in a decay of prediction accuracy (Lorenz et al., 2011). Neyhart et al. (2017) evaluated several methods for updating the training population in a long‐term GS and reported that using a smaller but more recent training population provided a slight advantage in prediction accuracy and genetic gain. Wientjes et al. (2022) suggested that a closely related training population is also important because trait genetic architecture is more similar to that of the selection candidates.

The pedigree‐based selection accuracy was much lower compared with that of the GS‐breeding programs. The improved selection accuracy in GS programs compared with pedigree‐based selection is due to the ability to predict the Mendelian sampling term. The Mendelian sampling is due to the fact that alleles transmitted from a parent to its offspring are sampled at random and therefore differ in each sibling (Werner et al., 2020). The genomic relationship matrix estimates relationships between siblings and other relatives more accurately than the pedigree relationship matrix because it is based on realized (identity‐by‐state) rather than expected relationships (Legarra et al., 2009). Because the Mendelian sampling term is included in GS, it is possible to determine whether siblings within a family are more or less related than expected, resulting in more accurate GEBV assignment and higher selection accuracy (Hill & Weir, 2011; Werner et al., 2020).

Selection accuracy in the early years of the future phase was lower for the ECT‐GS program compared with the other two GS programs. This is likely because fewer phenotypic records were used in the training population each year compared with Seed‐GSunc and Seed‐GSc programs. For comparison, only 90 records from the ECT stage in the ECT‐GS breeding program were used to update the training population compared with 500 from the ACT stage in the Seed‐GSunc and Seed‐GSc programs. The Seed‐GSunc breeding program had the highest overall prediction accuracy, confirming that training population size is an important factor in the development of GS‐breeding programs. This is consistent with the results of previous studies that showed that a large training population is required to accurately estimate marker effects (Combs & Bernardo, 2013; Zhang et al., 2017). However, it is still worth noting that the accuracy in the ECT‐GS program was not much lower despite the substantially reduced training records. This demonstrates the importance of high‐quality (multi‐year and multi‐location) phenotypic records in the training population.

Higher selection accuracy was observed when 25% of parents were replaced compared with when all parents were replaced. When all parents are replaced in each breeding cycle, a large shift in the LD pattern occurs and prediction within new families becomes more difficult. The shift in the pattern of QTL‐marker LD, if not captured, can lead to lower prediction accuracy (Lorenz et al., 2011). This also indicates that regular updating of the training population is important when many parents in the crossing block are regularly replaced. A stable decline in accuracy over time was observed in the Seed‐Ped and three GS programs. This was expected due to increasing genetic distance between training population and selection candidates and decreasing genetic variance as only a closed breeding population was considered (Gaynor et al., 2017).

### 4.3 Genetic variance and conversion efficiency

Maintenance of genetic variation is a critical component for long‐term breeding program sustainability and continued genetic progress. Selection causes changes in allele frequencies, genetic variances, and LD relationships between markers and QTL (Muir, 2007). The Seed‐Ped and the three GS programs suffered a substantial loss in genetic variance compared with the PS program. By applying pressure to SNPs associated with QTLs that largely contribute to the trait of interest, GS leads to rapid fixation of favorable alleles and high genetic gains in the short term (Wientjes et al., 2022). In the long term, GS increases the risk of losing rare favorable alleles or fails to increase the frequency of such alleles which reduces genetic variance, as may be observed in our study. Despite the much lower genetic gain, the loss of genetic variance of the Seed‐Ped program was similar to that of the GS programs. Similar results were reported by Wientjes et al. (2022), who argued that more segregating causal loci are lost through pedigree‐based selection likely because of stronger family‐based selection. Contrastingly, slower decrease in genetic variance observed in the PS is likely due to selection pressure being more evenly distributed across the genome which reduces the loss of segregating loci as a result of hitchhiking, apart from a much longer breeding cycle (Barton, 2000). Faster rate of loss in variance occurred especially in the early years of both the Seed‐Ped and GS programs due to sudden increase in selection accuracy. The increase in selection accuracy resulted in the rapid decrease in genetic variance under directional selection due to the build‐up of negative LD between causal loci (Bulmer, 1971).

Breeding programs differed in the conversion efficiency which measures the rate of variance lost and genetic gain produced. When 25% of the parents were replaced, the Seed‐Ped and GS programs had a lower conversion efficiency factor compared with the PS‐breeding program. This implies that selection based on PS is still an effective method because it converts genetic gain over genetic variance loss more efficiently but is too slow to produce the required genetic gains. When all parents were replaced, the conversion efficiency of the PS program decreased and increased slightly for the GS‐breeding programs. This is because the GS programs generated more genetic gain when all parents were replaced, although little difference in the loss of genetic variance was observed when 25 and 100% of parents were replaced. Overall, the lowest conversion efficiency was observed for Seed‐Ped, suggesting that GS is more sustainable than pedigree‐based selection in terms of maintaining future genetic gain.

The large reduction in genetic variance observed in GS programs can limit long‐term genetic gains in breeding (Cobb et al., 2019). The strategies that balance the rates of genetic gain and loss of diversity that could be implemented in tea‐breeding programs include optimal contribution selection (Yadav et al., 2020), optimal cross selection (Gorjanc et al., 2018), optimal contribution selection with branching (Santantonio & Robbins, 2020), optimal population value selection (Goiffon et al., 2017), and expected maximum haploid‐breeding value selection (Müller et al., 2018). As previously shown by Werner et al. (2020), crossing parents should be selected based on genomic predicted cross performance especially in the presence of dominance which was not considered in this study.

### 4.4 Simulation constrains and practical implementation of genomic selection in tea‐breeding programs

We used a practical commercial tea‐breeding program and its parameters as a basis to test the possible integration of GS. Our main constraints were low operating costs for breeding due to low labor wages and limited research resources. Kenya is one of the LMICs where wages are significantly lower compared with countries with advanced economies. For example, the average daily wage of a field worker in multinational tea companies in Kenya is ∼$5 (based on the collective bargaining agreement between workers and tea companies in Kenya), whereas the hourly wage in the UK is ∼$8–12 (according to glassdoor.co.uk). Our simulations have shown that implementation of GS is feasible despite these constraints. Although caution is still required in interpreting the results due to simulation limitations (see Gaynor et al. (2017) and Powell et al. (2020), our results provide useful conclusions for GS implementation in tea‐breeding programs:

1. Genotyping seedlings in nurseries and selecting the best parents based on GEBVs can greatly increase genetic gain by reducing generation interval and increasing selection accuracy.
2. Eliminating the early PT stage reduces the costs and shortens the breeding cycle by 3 yr. The saved cost can be reallocated for genotyping more seedlings in the nursery.
3. Elimination of the PT and ACT stages in the ECT‐GS program shortened the duration of the breeding cycle by 8 yr. However, genetic gains were lower compared with the other GS strategies because fewer training records were available for genomic prediction.
4. A $30,000 increase in the operating budget in the Seed‐GSunc resulted in only a small increase in genetic gain.
5. Genomic selection is cost effective in tea‐breeding programs when genotyping costs are $15 per sample or lower. Benefits are expected to increase as the cost of high‐throughput genotyping decreases in the future.
6. Consistent replacement of parents should occur in each breeding cycle to ensure continued genetic progress. However, optimal cross‐selection strategies may need to be considered.
7. In breeding programs where GS is not feasible due to financial and logistical constraints, pedigree‐based selection could be used as an alternative.

To ensure successful implementation of GS, breeders must ensure that the necessary facilities and equipment are available on site (e.g., freezers, sterile laboratories), that field technicians are appropriately trained, that genotyping and sample transport costs are identified, and that they collaborate with biometricians to optimize field testing and phenotype collection and to develop GS pipelines using appropriate statistical models (Covarrubias‐Pazaran et al., 2021). In our study, simple pedigree‐based and GS models were used for demonstration, but models that efficiently account for multiple harvests, as is the case in tea breeding, could be considered to obtain the most accurate predictions of genotype performance over time and across a range of environments (De Faveri et al., 2022).

### 4.5 Genomic selection for improvement of tea quality and practical implementation

Tea quality is another important attribute besides yield that was not considered in this study. Usually, tea quality is measured based on color, aroma, taste, mouthfeel of tea liquor, and appearance of dry tea (Zheng et al., 2016). These sensory attributes are derived from biochemical compounds present in fresh tea shoots, such as catechins, alkaloids, amino acids, and volatile compounds (Ivarsson et al., 2001). Traditionally, sensory evaluation using professional tasters has been the main method used to evaluate, grade, and determine the price of tea (Liang et al., 2003). Sensory evaluation is quick and practical to use. However, it requires skilled and experienced professional tasters (Liang et al., 2008). Sensory evaluation is subject to variation due to different preferences and mood of individual tasters (Sinija & Mishra, 2011). Hence, sensory evaluations should be complemented with chemical and physical analytical methods for identifying biochemical components associated with tea quality that are more objective, repeatable, and reproducible (Chen et al., 2015). These methods include liquid chromatography coupled with mass spectrometry (LC‐MS), nuclear magnetic resonance (NMR), near infrared (NIR) spectroscopy and chromatographic methods such as high‐performance liquid chromatography (HPLC) and gas chromatography (GC) (Yashin et al., 2015; Zheng et al., 2016). However, the use of most analytical techniques is currently still limited due to high purchase, maintenance, and expertise costs.

Genomic selection could also be used to improve selection for higher quality tea varieties (Lubanga et al., 2021). Currently, selection for tea quality occurs at a very late stage in the breeding program, and therefore many clones with favorable quality traits may have been discarded. Instead, data from professional testers and biochemical traits from analytical techniques in the late stages can be used to train the GS model in the nursery stage. Genotyped seedlings could be selected for multiple traits such as tea quality and yield simultaneously using a multi‐trait GS model framework and a selection index (Shahi et al., 2022). Selection for better tea quality could thus begin at the earliest stage, where variation in quality traits is greater. Simultaneous selection for tea yield and quality using GS and its effects on the overall genetic gain are currently unclear and need further investigation.

## 5 CONCLUSION

Our study provides insights into the optimal implementation of GS in tea breeding under constrained operating costs in LMIC. Although the ECT‐GS program seems more desirable because of its short breeding cycle (8 yr) and competitive genetic gains, we believe that breeders may be reluctant to allow radical changes to their breeding programs and therefore, the Seed‐GSc program is a more attractive strategy. The Seed‐GSc program has the same budget as the PS program and retained the multi‐year and multi‐location clonal trials that provide essential information for accurate genomic prediction. Most tea‐breeding programs have pedigree information that is rarely used to predict the performance of promising genotypes. Our simulation showed that pedigree‐based selection had higher genetic gain compared with PS and should therefore be considered when direct transition to GS is not possible or when GS cannot be implemented. The results shown in this paper are not only applicable to tea‐breeding programs but also to other perennial‐ and clonal‐breeding programs.

## AUTHOR CONTRIBUTIONS

Nelson Lubanga: Conceptualization; Formal analysis; Funding acquisition; Investigation; Methodology; Project administration; Writing – original draft; Writing – review & editing. Sean Mayes: Writing – review & editing. Festo Massawe: Writing – review & editing. Gregor Gorjanc: Conceptualization; Supervision; Writing – review & editing. Jon Bančič: Conceptualization; Methodology; Writing – original draft; Writing – review & editing.

## CONFLICT OF INTEREST

The authors declare no conflict of interest.

## Data Availability

The R scripts to perform the simulations analyses using AlphaSimR are publicly available on the GitHub repository: https://github.com/HighlanderLab/nlubanga_teabreeding.
